# Online classified adverts reflect the broader United Kingdom trade in turtles and tortoises rather than drive it

**DOI:** 10.1371/journal.pone.0288725

**Published:** 2023-07-13

**Authors:** Jon Bielby, Andy Ferguson, Matthew Rendle, Kirsten M. McMillan

**Affiliations:** 1 School of Biological and Environmental Sciences, Liverpool John Moores University, Liverpool, England; 2 Lincolnshire Wildlife Park, Friskney, Boston, Massachusetts, United States of America; 3 Association of Zoo & Exotic Veterinary Nurses, Market Harborough, United kingdom; 4 Dogs Trust, London, United kingdom; University of Liverpool & International Livestock Research Institute (ILRI), UNITED KINGDOM

## Abstract

Online sales are increasingly a route by which exotic animals are sold in the global pet trade. There are numerous types of online platforms and transaction types, and dedicated classified advertisement sites are a popular means of buying and selling animals. Despite their large and increasing use, we have a relatively poor understanding of the number of, and taxonomic variation in, the animals sold online. This information may be key in efforts to optimise the welfare of the animals being sold, and the ethics and sustainability of the trade via that platform. To fill this knowledge gap, we monitored and analysed the advertisements of chelonians (turtles and tortoises) placed on one of the United Kingdom’s largest dedicated classified ads sites, www.pets4homes.co.uk, over the course of a year, from July 2020 until June 2021. We analysed temporal, taxonomic, and advertiser related trends in the volumes of advertisements placed and compared the prices and the sentiment of language within adverts for different species. We found that the species advertised, the prices requested, and infrequent use of the site by most advertisers is consistent with most adverts being for animals being resold by casual users. Further, we found that turtles were consistently advertised for lower prices and in multiples than tortoises, and that the language with which they were advertised was less positive. We conclude that on this website the online trade reflects the broader trade, rather than drives the sales of chelonians in the UK, and that any interventions aiming to improve welfare and sustainability would be better placed earlier in the supply chain.

## Introduction

Around 1.2 million UK households keep a reptile of some kind, which is approximately the same number that keep more traditional pets such as rabbits or domestic fowl [1]. The animals that meet this demand vary in their provenance (wild/farmed/captive-bred), their conservation status (threatened/non-threatened), the legality of the transactions involved (legal/illegal), and whether the animals are transported across international boundaries or not (in-country/imported). The point-of-sale of these animals also varies, including licensed shops [2], face-to-face sales at trade shows [3], dedicated online platforms [4], and social-media sites [5–7]. Given the variation in these elements of the reptile supply chain, the methods of monitoring and, where necessary, intervention will need to be flexible and targeted.

One aspect of the reptile pet supply chain that appears to be increasing is internet trade [7]. This route of transactions may occur via several different types of sites. These include dedicated e-commerce sites, which generally involve the transaction between seller and buyer taking place online; classified advertisements, which connect seller and buyer who complete the transaction offline; and social media (see reference [7] for a review of the types of websites relevant to wildlife trade generally). These categories of sites vary in numerous ways including legal requirements and regulations surrounding advertisement and sale, payment methods, and scale of operations. Research into the online pet trade suggests that potential risks to animal welfare and biodiversity of conservation may exist, and that online sites may be a useful source of data on the pet trade. For example, social media sites and interviews with users have provided valuable information on the demographic and geographic nature of pet owners [8,9] as well as the taxonomic diversity of its constituent species [10]. Research into the online trade has also highlighted the presence of illegally advertised species [11] and the attitudes of actors in the broader trade on the risk of disease spread it may pose [12]. Social media sites are a common route for sales of exotic pets, and platforms such as Facebook and Instagram have formed the basis of data collection for several studies relating to the trade in exotic pets from a range of taxa [4,9,13–16]. In response to concerns over the welfare of animals being sold and the difficulties of policing illegal online trade, Facebook banned the sale of live animals in 2017 and in late 2020 reiterated these guidelines (e.g., [17]). Whether these changes will alleviate the problem entirely or drive the supply chain further into the Deep Web (content not indexed by standard web search-engine programs e.g., chat messaging and private content on social media sites) or the Dark Web (unindexed sites or those requiring specialist software to gain access [7]) remains to be seen. Another possibility is that much of the trade formerly occurring via Facebook (and/or other social media sites making similar efforts) will remain on the surface web and move towards other online platforms, such as e-commerce sites, and online classifieds.

Websites of classified advertisements for exotic pets are generally more amenable to self-policing than social-media sites, but they are not without their own challenges and process gaps. These include the lack of suitable trading standards, which may largely be designed for physical sales (e.g., shops); difficulty of monitoring and reporting illegal activity [18]; and the possibility of cheating the online system by, for example, misrepresenting or fabricating descriptors attached to the advertisements e.g., species or product being sold [19], or advertising certain species illegally [11]. For example, Sung *et al*. [4] found that species sold, conservation status, and CITES status, all varied according to point-of-sale: whilst trade on social media contained the most threatened and CITES-listed species, online forums selling animals still contained more potentially illegal species than physical shops and markets. Similarly, comparisons of expos, physical shops, and online adverts in Japan highlighted the likelihood of illegal trade, with notable differences in species diversity and species conservation status between sales routes, and the same advertisers promoting different species online compared to those in their physical shops [20]. So, while online classified advertisements sales offer some solutions for reducing concerns related to trade in exotics, they may still have issues related to legal compliance and best-practice both compared to social media and potentially to other routes such as dedicated e-commerce sites.

Taxa covered in studies of online sales are diverse, but a large number have focussed on the trade in chelonians, i.e., tortoises and turtles, which appear to have been negatively affected by the pet trade in several ways [21,22]. At point of collection, the demand for certain species has been linked to population decline and, in some cases, local extinction [23]. At the other end of the supply-chain, the large numbers traded, husbandry challenges, and complex life histories, have resulted in ethical concerns for the keeping of some more challenging species, while other more generalist species have become highly invasive in some regions due to the release of unwanted pets into the wild [24–26]; for example, individuals of *Trachemys* species are now found to have a near global distribution, are listed as one of the top 100 invasive species worldwide, and are illegal to trade in many countries including the UK [27]. The frequency with which turtles occur as non-native species perhaps reflects the volume in which they’re traded, the difficulty of their captive husbandry, and their ease-of purchase, all of which are high compared to other reptiles, including terrestrial chelonians (tortoises). Further, at multiple points in-between the two ends of the supply-chain, chelonians may experience compromised welfare due to factors relating to capture, transport, sales, and post-sale husbandry [28]. Clearly, understanding the type, provenance, status, and number of animals sold via online classified advertisements would be a useful tool in identifying whether any of the issues outlined above need addressing via within-country legislation and management.

Sales platforms (online or in person) may vary greatly in the nature of advertisers (e.g., breeders, shops, casual), type of the animals being advertised (rarities, specific colour morphs, commonly kept species), and price at which they are sold. If a platform were focussed toward specialist breeders or importers, we may predict advertisements to be dominated by a relatively small number of advertisers aiming to sell a diverse range of species, including those that are less common in the trade, at a higher price. In contrast, if casual sellers placed the most advertisements, we might predict a smaller range of species, sold for lower prices from a larger pool of sellers. Further, if the supply of animals being advertised were captive-bred we might expect there to be seasonal trends for at least a subset of species, whereas casual users may place their advertisements more randomly throughout the year. Of course, platforms are likely varied and fall between these extremes, but at present we have little empirical understanding of the relative use of general classified advertisement sites by different types of sellers-and-buyers. This information is essential, if we are to better understand where on the supply chain they sit, and what role they play in the chelonian trade. In this study we present analyses on a year’s chelonian advertisement data from one of the UK’s most used, dedicated sites for classified pet adverts, www.pets4homes.co.uk. We aimed to answer the following questions:

What taxonomic and temporal patterns exist in the advertisements placed?

What is the relative use of the site by different types of sellers (e.g., frequent, intermediate, and casual)?

How does listed cost per animals vary among species-types?

How does language use within advertisements vary between user-category and species-type?

By answering these questions and considering interactions between them (e.g., does species-type vary with seller-category?), we aim to obtain quantitative estimates of the users of this site, species advertised, and drivers of the online chelonian supply.

## Methods

### Data collection and definitions

All data wrangling and analyses were conducted in the software R [29]. Specifically, the following packages were used for data extraction and wrangling: *tidyverse* [30], *rvest* [31], *stringr* [32] and *dplyr* [33], and *ggplot2* [34] was used for producing figures.

### Data collection and ethics statement

We used a covert observational approach on content in the public domain. The study received approval from the Liverpool John Moores University ethics committee. As outlined below the nature and type of data collected, and scale of analyses conducted meant that no potentially identifiable human images or data were presented or collected in this study, and no individuals were recognisable throughout the data collection or analysis process. As with similar studies previously, the covert approach was necessary because there currently exist no means of getting prior informed consent for a study of this kind [4,35].

Data were collected daily from July 1st 2020 to June 26^th^ 2021 from dedicated pet classified advertisement (advert) site www.pets4homes.co.uk. For each advert placed on chelonians between those dates inclusive, we collected information on date the advert was first posted, a unique seller ID, price placed on the advert, species-type advertised (as defined by the website), short description of the animal(s) and, where present, additional products (e.g., heat lamps, aquariums) being sold. Using the combination of these data, a unique identifier was attached to each advert.

We categorised animals into groupings that we refer to here as ‘species-types’, because they do not represent true species or breeds. Based on preliminary data exploration, all adverts were placed into one of 35 species-types (see S1 Table for a full list and associated advert frequency). The allocation of an advert to a species-type was based on the presence of certain words within the advert description identified using the *stringr* library in R and were further validated by manual checking. However, some level of misidentification may have occurred as we relied on advert descriptions for categorisation alone i.e., species-type was not inferred/identified from associated photographs. Species types that were advertised fewer than 50 times over the course of the year were removed from the dataset before analyses; this avoided technical problems with multilevel *post hoc* tests which occurred due to an unbalanced dataset.

Using the advert description, we determined whether multiple animals were being sold together in the same advert. To ascertain whether adverts contained one or more individuals for sale together, each advert description was checked manually. A binary variable was developed: presence of individual animal (0) or multiple animals (1). All individuals within an advert were assumed to be of the same species-type, unless stated otherwise. In some cases, individuals could be bought separately on request, but the original advertisement stated they were on sale together.

Hermann’s tortoise requires a CITES Article 10 license to be sold in the UK. To identify whether adverts contained the correct legal information regarding this species-type, we manually searched the advert description free-text for any of the following terms: “cites”, “licen*”, “permit”, “a10”, and *microc*”.

For our presented analyses and results, the unique seller ID code was utilised to place sellers into categories. Inclusion within seller-category was dependent upon the number of adverts placed, by seller, over the study period. Sellers were considered (1) ‘casual’, if they had placed one or two adverts within the year, (2) ‘intermediate’, is they had placed three or four adverts over the course of the year, and (3) ‘frequent’ if they had posted five or more adverts over the year. The model building process for adverts placed per month was also conducted using the frequency of adverts placed per seller over the course of the year, rather than the categories described above (i.e., we totalled the number of adverts placed by those sellers who only posted once per year, those who posted twice per year, those who posted three times per year, and so on). The results and relative model performance were qualitatively the same for both data treatments, and so for analytical purposes we focus here on the analyses and results using the categories of seller type, which provided a more even balance of seller posting frequency and species type.

### Statistical analysis

To investigate the taxonomic and temporal patterns regarding frequency of advert posting the number of adverts placed per month was the response variable within generalised linear models with Poisson errors, constructed using the R package lme4 [36]. Predictor variables included:month advert was placed, species-type, and seller-category. We compared model performance using AIC, considering models within 6 units of each other to perform equally well [37]. All combinations of individual variables were included in the model building process, and two interactions terms were also included: month advert was placed and species-type, and seller-category and species-type. Goodness of fit of the model was calculated using the equation: 1—residual deviance/null deviance. Using the best performing model, we applied the function glht() in the *multicomp* library [38] to determine where differences in levels of factorial variables existed (month advert placed, species-type, seller-category). To identify whether certain species-types were more likely to be advertised by different seller-categories, we used binomial tests within a species-type to compare the proportion advertised by each seller-category with the background proportion of total adverts placed by that seller-category across species-types.

Next, we modelled the probability that an advert would be selling more than one individual, using generalised linear models with binomial errors i.e., individual animal (0) or multiple animals together (1). Predictor variables included: species-type, seller-category and an interaction between them, in order to ascertain whether certain types of sellers would be more likely to sell multiple individuals of certain species-types together. The month advert placed was also included as a predictor in this model, as this could be another source of variation regarding the number of animals included for sale within an advert. Model performance, goodness of fit and differences between levels of factorial variables were assessed and calculated as above.

To assess whether the listed cost per advert varied according to species-type, we excluded adverts containing more than one individual as well as those containing equipment, such as housing or lighting. Remaining data included 1211 adverts, representing the 10 most frequently advertised species-types. Given the error structure of the data, we compared prices using a Kruskal-Wallis non-parametric test with the listed price of the advert as our response variable, and species-type as the grouping factor. Dunn’s test was used as a *post hoc* test to identify where any significant differences lay in the listed price per advert, among species-types.

### Sentiment analysis

Sentiment analyses were conducted using the aforementioned *tidytext* package, implementing two general purpose lexicons: *afinn* [39], and *nrc* (syuzhet [40]). Both contain many English words and are based on tokens, i.e., single words, which are assigned scores for positive/negative sentiment. The *afinn* lexicon awards words with a score of -5 to 5, with negative scores indicating negative sentiment and positive scores indicating positive sentiment. The *nrc* lexicon categorizes words into ten divisions: positive, negative, anger, anticipation, disgust, fear, joy, sadness, surprise, and trust. Both lexicons were used here to investigate variation in measures of sentiment among the three seller-categories and among the 10 most frequently advertised species-types.

## Results

### Summary of dataset

A total of 2856 adverts were placed over the study period. 272 of those represented 24 species-types that each featured in fewer than 50 adverts over the course of the year, and were therefore excluded from the analyses. This left 2584 adverts with a mean of 7.07 adverts per day (s.d. = 3.18), representing 10 species-types. All higher-level quantitative analyses were conducted on the full dataset that included these species-types and adverts containing them, and the results were qualitatively the same as those presented here. All species-types that were advertised are presented in S1 Table.

The best supported model of the number of adverts placed per month explained 90.2% of the variation, was 78 AIC units lower than the second best supported model, and contained terms for the seller-category, species-type, month advert placed, and an interaction between seller-category and species-type (Table 1 for AIC comparisons among models; S2 Table for the analysis of deviance output of the best model).

**Table 1:** Performance of models of number of adverts placed per day over the course of the study. Models are ranked by AIC and the top model and those receiving equal support are highlighted in bold. Models within 6 AIC units of the top model were deemed to receive equal support.

Model	d.f.	AIC	ΔAIC	Goodness of fit
Species-type + Month advert placed + Seller-category + Species-type*Seller-category	**41**	**1472.47**		**0.902**
Species-type + Month advert placed + Seller-category	23	1550.52	78.05	0.861
Species-type + Seller-category	12	1570.87	98.40	0.845
Species-type + Month advert placed + Seller-category + Species-type*day of year	122	1655.77	183.30	0.894
Species-type + Month advert placed	21	2671.96	1199.48	0.452
Species-type	10	2693.98	1221.50	0.436
Seller-category + Month advert placed	14	2843.50	1371.02	0.385
Seller-category	3	2863.74	1391.26	0.369
Month advert placed	12	3844.64	2372.17	0.020

**Fig 1:**
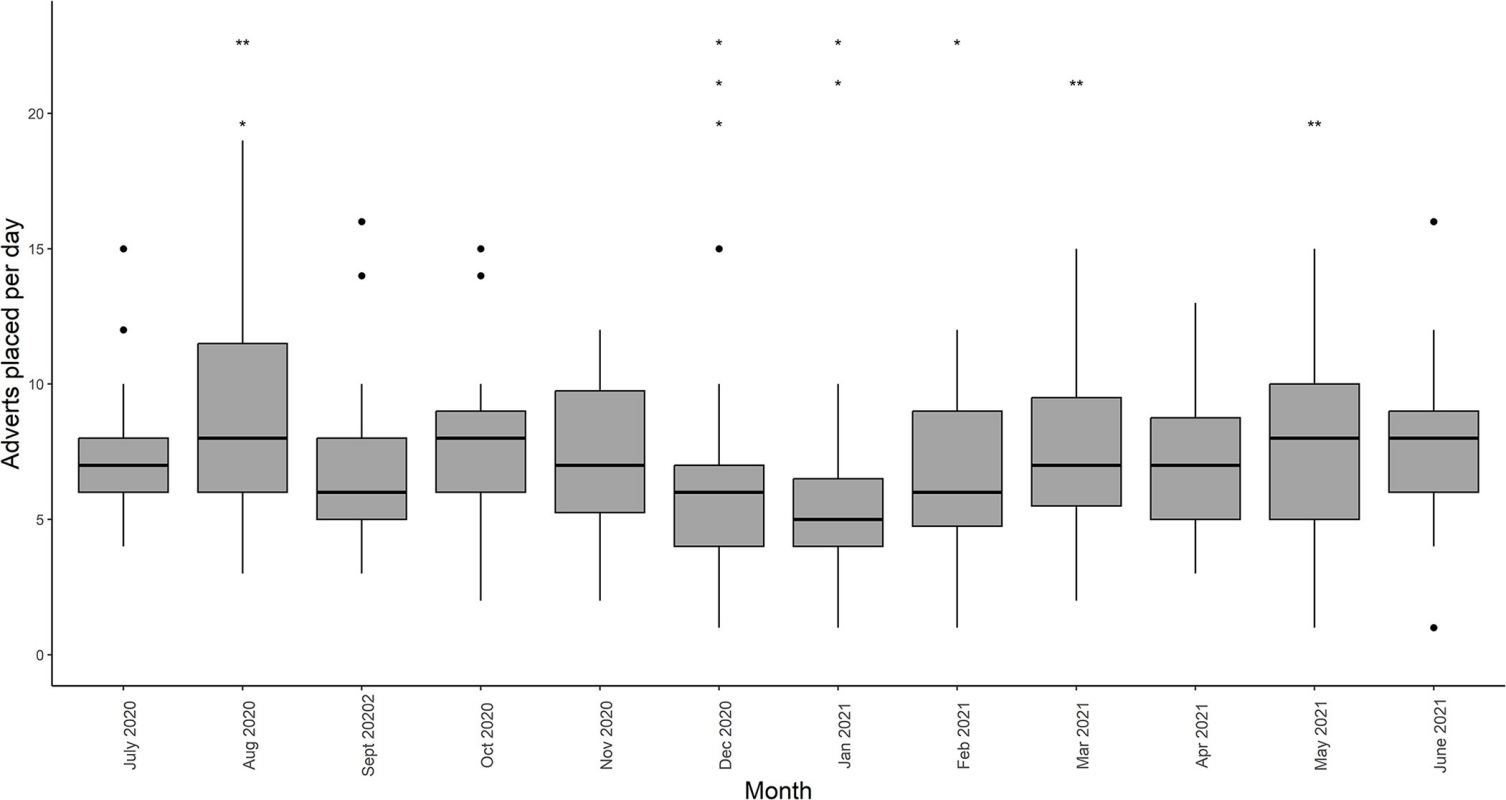
Advertisements placed per day for each of the 10 most frequently advertised species-types over the study period. Asterisks above box-plots indicate comparisons where significant differences occur. For each horizontal layer the ** denotes the month with a significantly higher rate of advertisements placed per day compared to those with *. For example, August 2020 has a significantly higher rate per day than December 2020, January 2021, and February 2021.

Temporal variation in the number of adverts posted per month was largely related to a lull in the winter months of December, January, and February (Fig 1). *Post hoc* tests suggested that January (mean = 5.39, s.d. = 2.28) had significantly fewer adverts posted than March (mean = 7.67, s.d. = 3.07), May (mean = 7.62, s.d. = 3.59) and August (mean = 8.65, s.d. = 4.23); December (mean = 6.03, s.d. = 3.02) had fewer than March and August; and February (mean = 6.46, s.d. = 3.02) had fewer than August (S3 Table).

**Fig 2:**
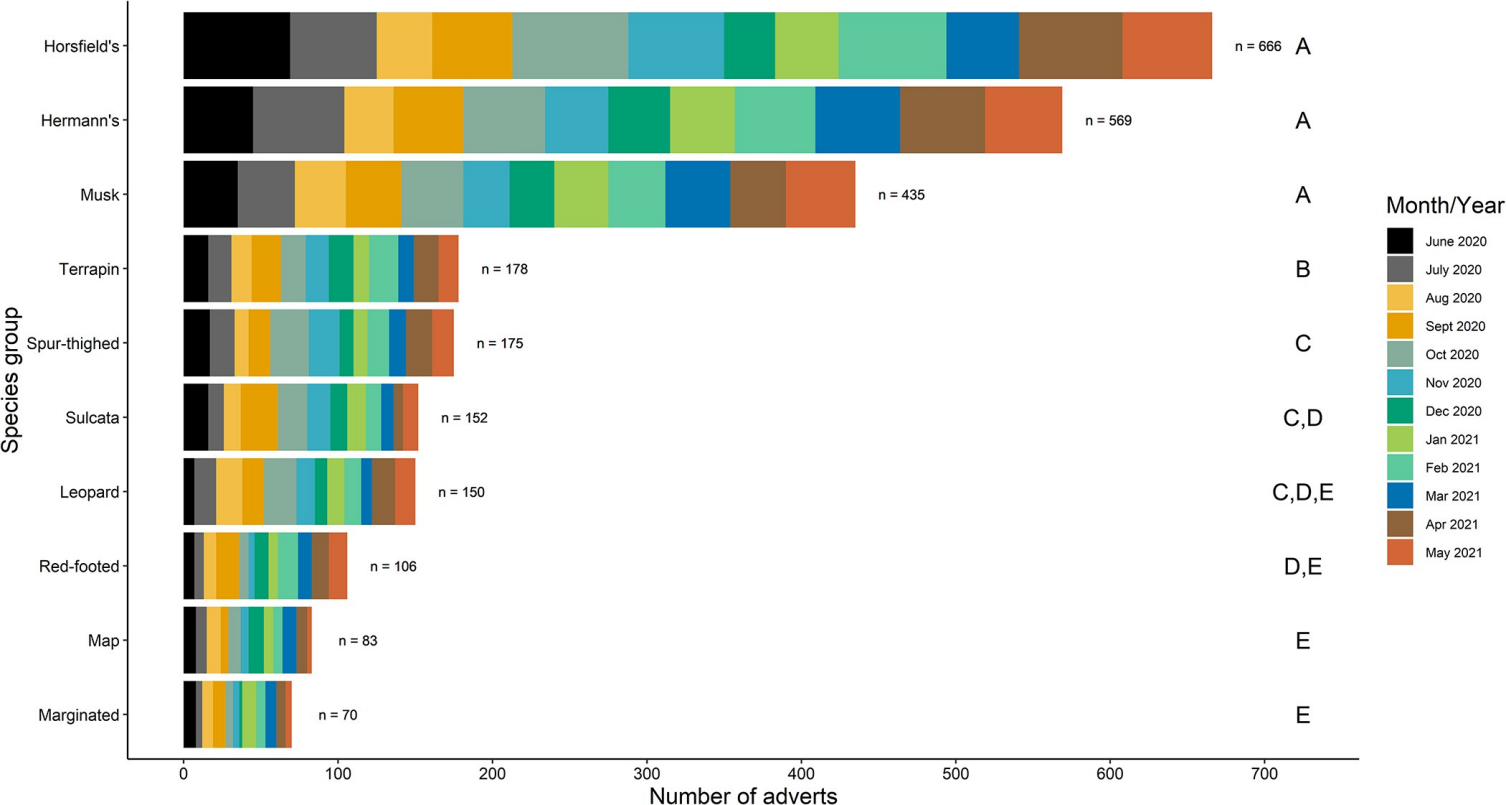
Total number of each of the 10 most frequently advertised species-types (number of advertisements are at the end of each bar). Colours represent the number of adverts of each species-type for each month of the study. Bold letters A-E represent where significant differences lie in advertisement frequency among species-types based on multilevel comparison of multivariable glm output. Comparison of the multivariable glm output means simply comparing the size of the bars does not represent the difference between species, as other factors are not accounted for.

Taxonomically, there were clear differences between the number of adverts placed among species-types, with Horsfield tortoises (*Agrionemys horsfieldii*), Hermann’s tortoises (*Testudo hermanni*), and musk turtles (*Sternotherus* spp.) being most frequently advertised, representing 65.4% of all adverts placed (totals of 666 (25.7% of total), 569 (22.9% of total), 435 (16.8% of total) advertisements respectively). *Post hoc* tests (S4 Table) suggest that these three species-types were advertised in significantly more adverts than any other species-type. Other differences between adverts posted existed between species-types as highlighted in S4 Table and Fig 2. The lack of importance of the interaction between species-type and month advert placed is highlighted by the relatively even distribution of adverts per month across all species-types (Fig 2). Of the 569 adverts selling Hermann’s tortoises, only 16.2% (n = 92) included text outlining legal compliance regarding licensing of selling the species (e.g., text confirming that they were microchipped or had a CITES A10 license).

**Fig 3:**
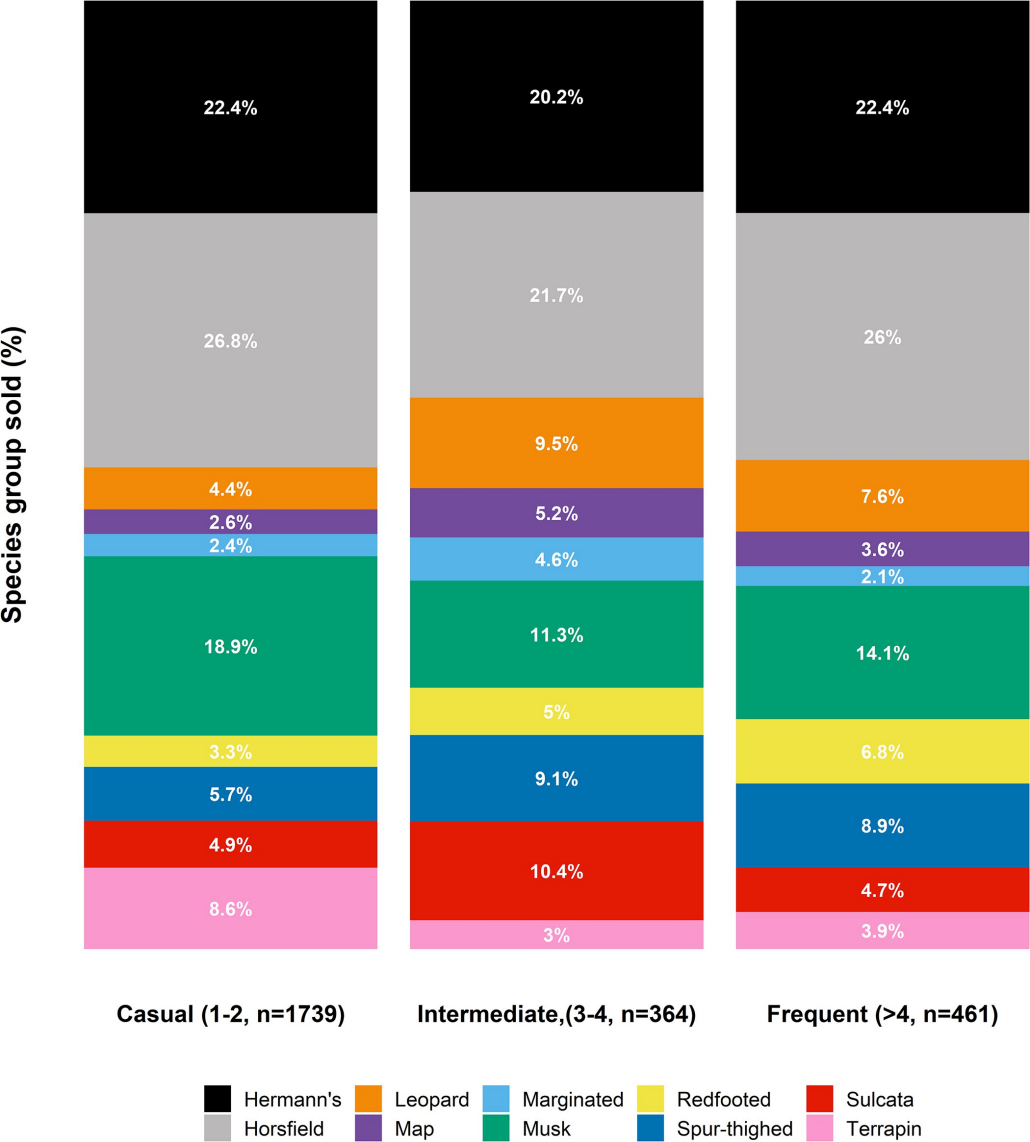
Plot of the proportion of adverts comprised of the different species-type per different category of seller. Colours represent species-type. Sellers were considered (1) ‘casual’, if they had placed one or two adverts within the year, (2) ‘intermediate’, is they had placed three or four adverts over the course of the year, and (3) ‘frequent’ if they had posted five or more adverts over the year.

*Post hoc* tests suggest that the majority of adverts were posted by casual sellers i.e., those who posted only once or twice within the 12 months study period (n = 1739 adverts), and that they posted significantly more than intermediate (n = 384 adverts) or frequent sellers (n = 461 adverts) (S5 Table; The frequency of sellers placing each number of adverts during the year can be seen in S6 Table). The interaction between seller-category and species-type included in the best performing model of number of advertisements placed per day, is highlighted by the disproportionately large absolute number of adverts for the most common species-types, i.e., Hermann’s tortoises, Horsfield tortoises, Musk turtles, posted by casual users. Once the absolute number of posts is accounted for, the proportion of advertisements per species-type is broadly consistent among the different type of sellers (Fig 3). However, notable differences exist between seller-categories for certain species-types, with frequent users having a higher proportion of their advertisements including sulcatas (Casual 4.9%; Intermediate 4.7%; Frequent 10.4%), spur-thighed tortoises (Casual 5.7%; Intermediate 8.9%; Frequent 9.1%), and leopard tortoises (Casual 4.4%; Intermediate 7.6%; Frequent 9.5%), whereas casual users present a higher proportion of musk turtles (Casual 18.9%; Intermediate 14.1%; Frequent 11.3%) and terrapins (Casual 8.6%; Intermediate 3.9%; Frequent 3.0%).

**Fig 4:**
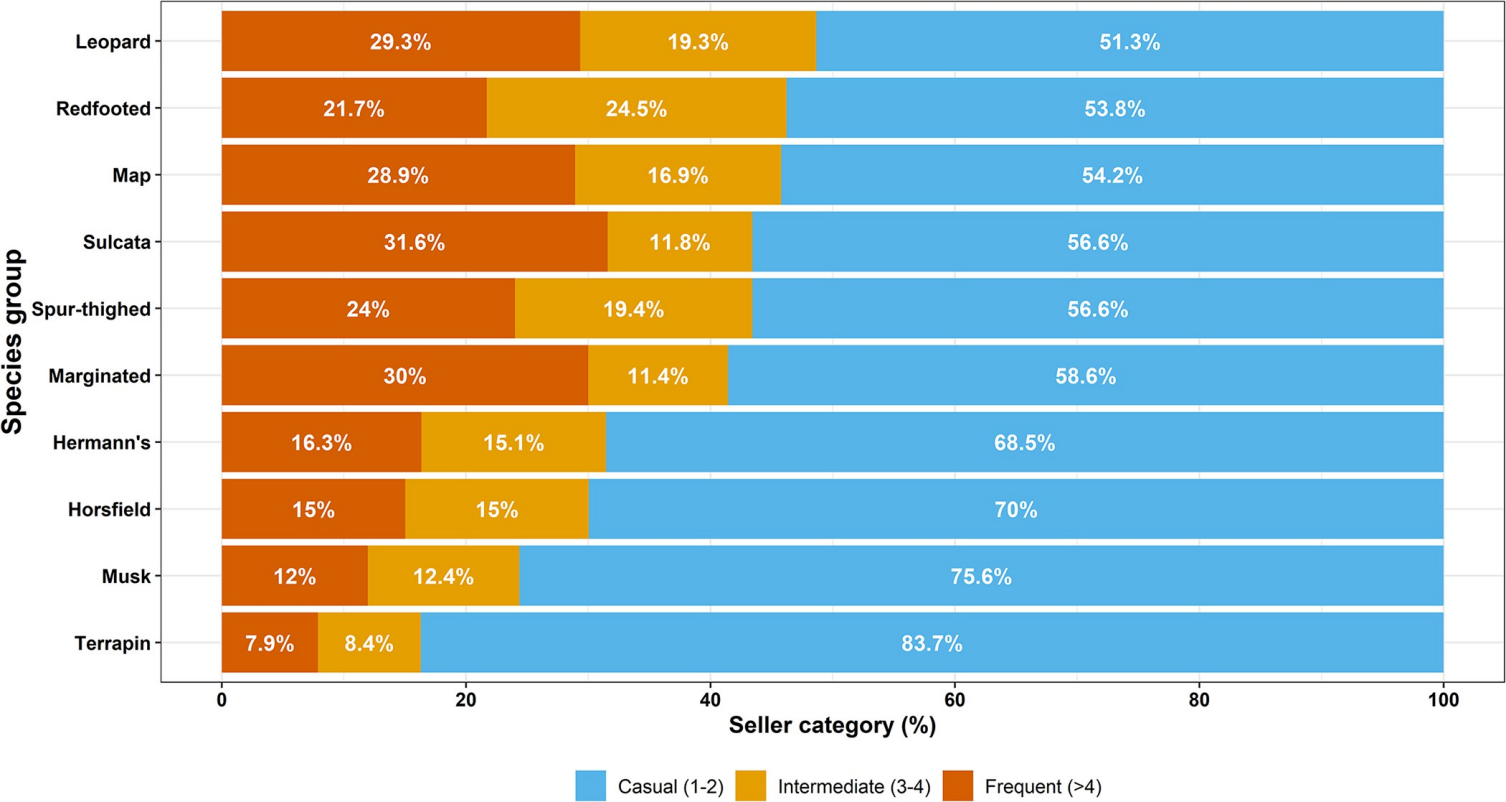
The percentage of adverts placed by each seller-category within each of the top ten most frequent species-types. Sellers were considered (1) ‘casual’, if they had placed one or two adverts within the year, (2) ‘intermediate’, if they had placed three or four adverts over the course of the year, and (3) ‘frequent’, if they had posted five or more adverts over the year.

Looking in more detail at the proportion of seller-categories placing adverts per species-type, highlights some strong trends in the data (Fig 4). Binomial tests indicate that terrapins and musk turtles both had a higher proportion of adverts placed by casual users than we would expect by chance (terrapins, χ^2^ = 19.98, df = 1, p-value <0.001, musk turtles χ^2^ = 11.60, df = 1, p-value <0.001). The next two species-types most commonly advertised by casual users were not significantly different from the background proportion for each seller-category (Horsfield tortoise χ^2^ = 1.61, df = 1, p-value = 0.203, Hermann’s tortoise χ^2^ = 2.74, df = 1, p-value = 0.601).

### Analysis of adverts containing multiple individuals

**Fig 5:**
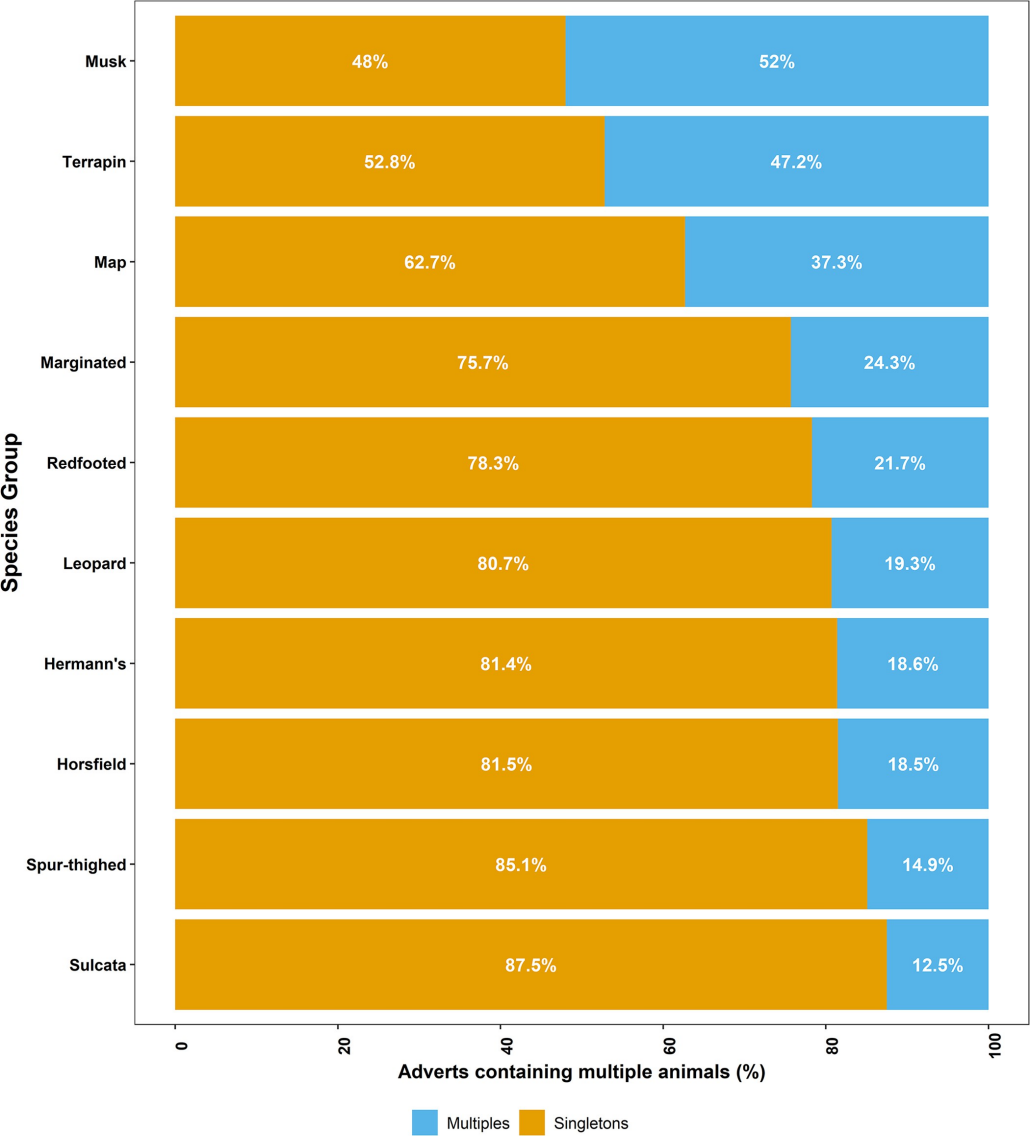
Proportion of advertisements containing multiple individuals for sale together across the top ten most commonly advertised species-types.

Of the 2854 adverts posted during the study period, 74.5% advertised a single animal (n = 1900), 21.6% advertised two animals for sale (n = 559), 3.0% advertised three (n = 78), 1.4% advertised four (n = 37), 0.2% advertised five individuals (n = 6), 0.03% advertised six (n = 1), 0.03% advertised seven (n = 1), and 0.07% advertised eight individuals(n = 2). The best performing models for explaining the probability of an advert containing more than one individual for sale, contained species-type and seller-category (Table 2) and explained 9.5% of the deviance in the response variable: i.e., certain species-types are more commonly advertised with multiple individuals for sale together (Fig 5, S7 Table). P*ost hoc* tests suggest that turtles rather than tortoises were more commonly sold in multiples. Specifically, musk turtles, terrapins, and map turtles were significantly more likely to be advertised for sale as multiples than the tortoise species Hermann’s, Horsfield, spur-thighed and sulcata. Additionally, musk turtles, which were the species-type most often sold in multiples, were significantly more likely to be done so than leopard tortoises.

**Table 2:** Performance of models of number of individual animals for sale in a single advert. Models are ranked by AIC and the top model and those receiving equal support are highlighted in bold. Models within 6 AIC units of the top model were deemed to receive equal support.

Model	d.f.	AIC	ΔAIC	Goodness of fit
**Species-type + Seller-category**	**12**	**2726.66**		**0.095**
Species-type + Month advert placed + Seller-category	23	2743.57	16.71	0.097
Species-type + Seller-category + Month advert placed + Species-type*Seller-category	41	2759.63	32.97	0.103
Species-type + Month advert placed	21	2777.35	50.69	0.084
Species-type + Seller-category + Month advert placed + Species-type*Month advert placed	122	2807.19	80.53	0.141
Seller-category	3	2936.73	210.73	0.019
Seller-category + Month advert placed	14	2952.97	226.31	0.020
Month	12	3005.01	278.35	0.002
Species-type + Seller-category + Species-type*Seller-category	30	3030.97	304.31	0.118
Species-type	10	3034.86	308.20	0.94

**Fig 6:**
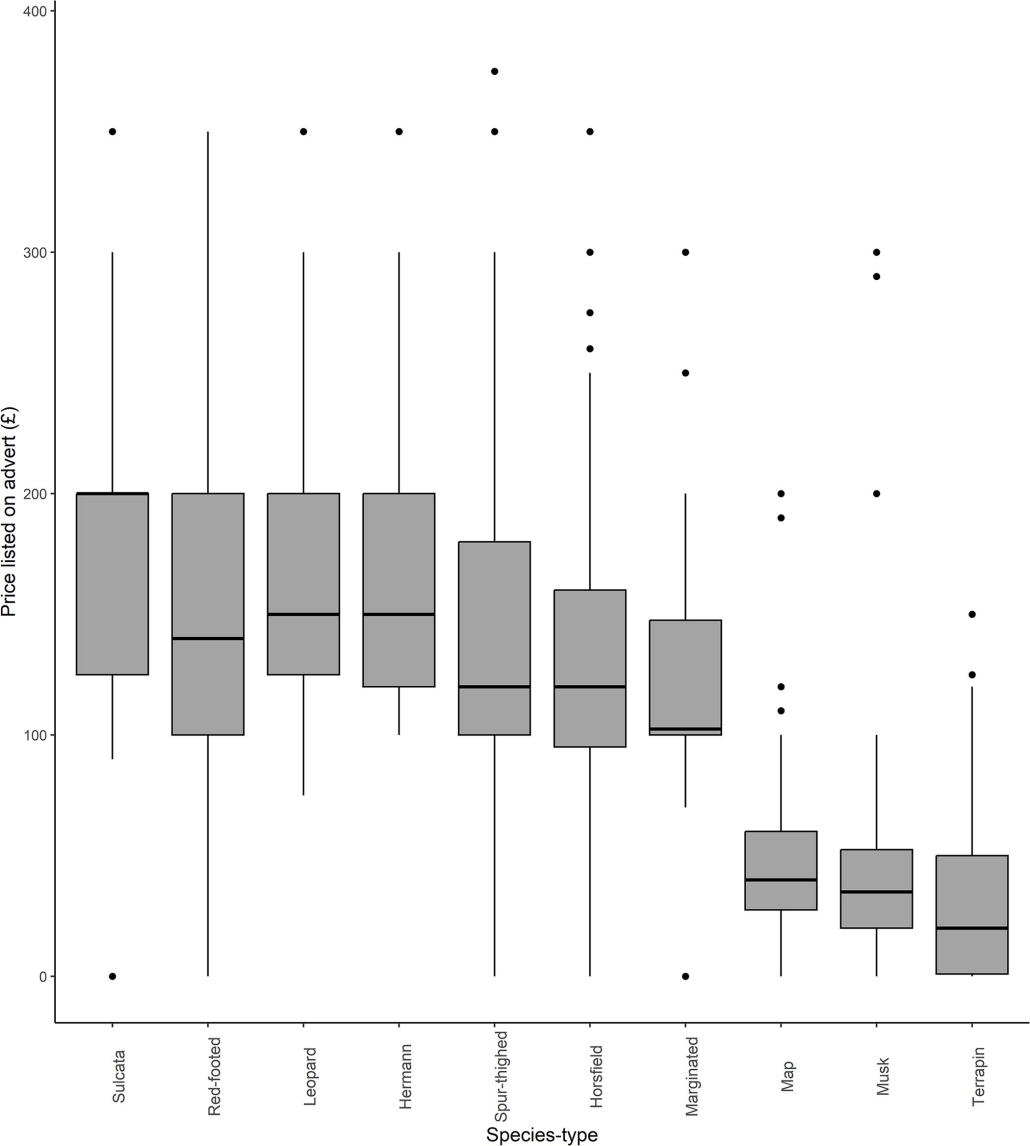
Prices listed in advertisements of the ten most commonly advertised species-types. Pairwise significant differences between species types are shown in Table 3.

Listed cost per advert varied significantly among species-type (Kruskal-Wallis chi-squared = 485.28, d.f. = 9, p<0.001, Fig 6). *Post hoc* Dunn’s test (Table 3) suggested that the most commonly sold turtles: map turtles (mean = £50.03, s.d. = 44.68) musk turtles (mean = £43.38, s.d. = 43.75), and terrapins (mean = £33.49, s.d. = 39.41) were significantly cheaper than all other listed species-types and tended to have less variation in price compared to other species-types (see Fig 6). Of the other most commonly listed species, Horsfield tortoises (mean = £139.11, s.d. = 72.76) were significantly less expensive than Hermann’s (mean = £163.72, s.d. = 90.99), leopard, red-footed, and sulcata tortoises, while Hermann’s were significantly less expensive than sulcata.

**Table 3:** *Post hoc* Dunn’s test results on the price of animals listed in the advertisements.

	Hermann	Horsfield	Leopard	Map	Marginated	Musk	Red-footed	Terrapin	Spur-thighed
Horsfield	**0.019**								
Leopard	0.459	**<0.001**							
Map	**<0.001**	**<0.001**	**<0.001**						
Marginated	0.598	1.000	0.059	**<0.001**					
Musk	**<0.001**	**<0.001**	**<0.001**	1.000	**<0.001**				
Red-footed	0.486	**0.002**	1.000	**<0.001**	0.060	**<0.001**			
Terrapin	**<0.001**	**<0.001**	**<0.001**	1.000	**<0.001**	1.000	**<0.001**		
Spur-thighed	1.000	0.605	0.243	**<0.001**	1.000	**<0.001**	0.265	**<0.001**	
Sulcata	**<0.001**	**<0.001**	**0.029**	**<0.001**	**<0.001**	**<0.001**	0.158	**<0.001**	**<0.001**

**Fig 7:**
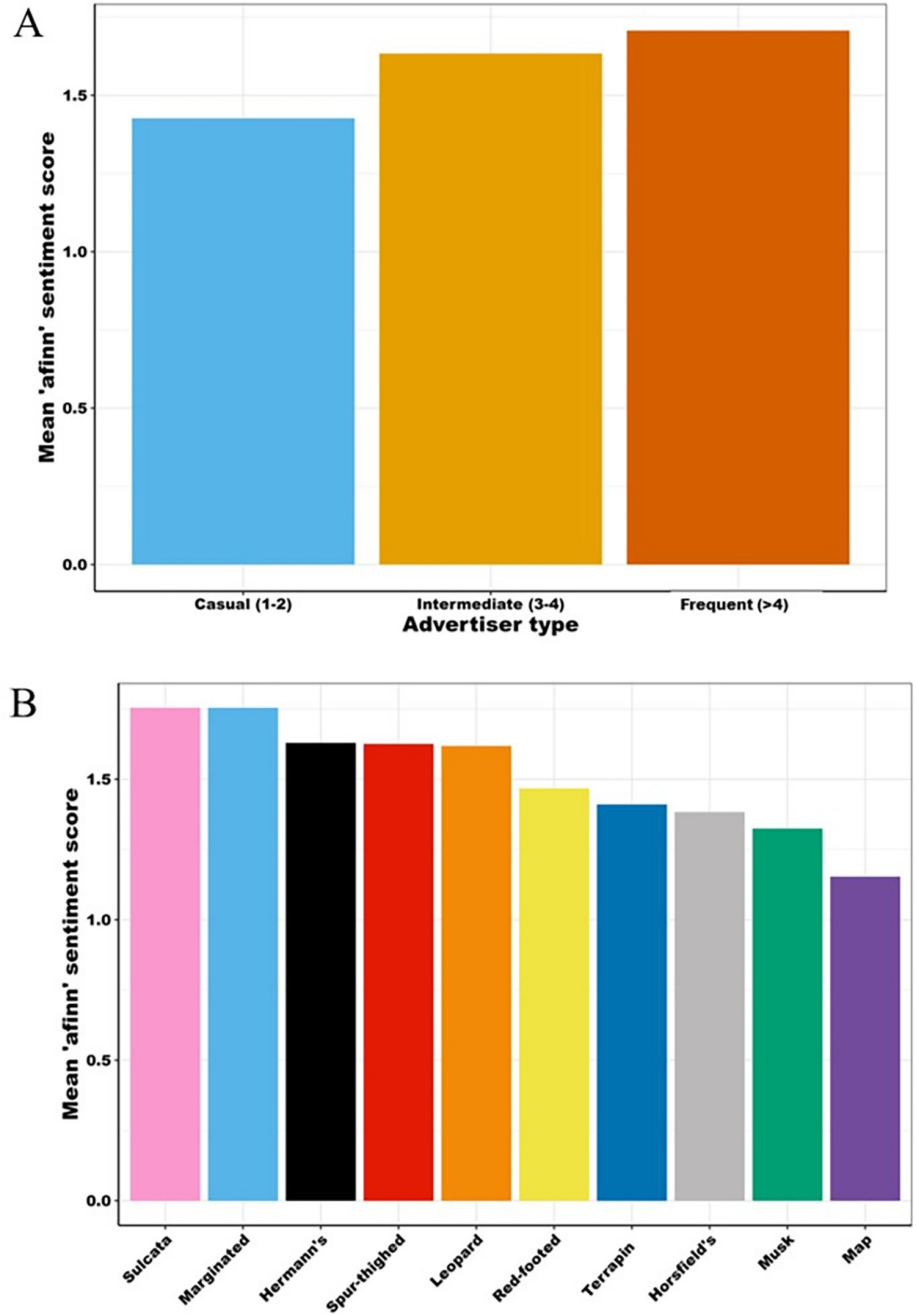
Mean *afinn* sentiment scores in advertisements categorised by A) seller-category, and b) species-type.

**Fig 8:**
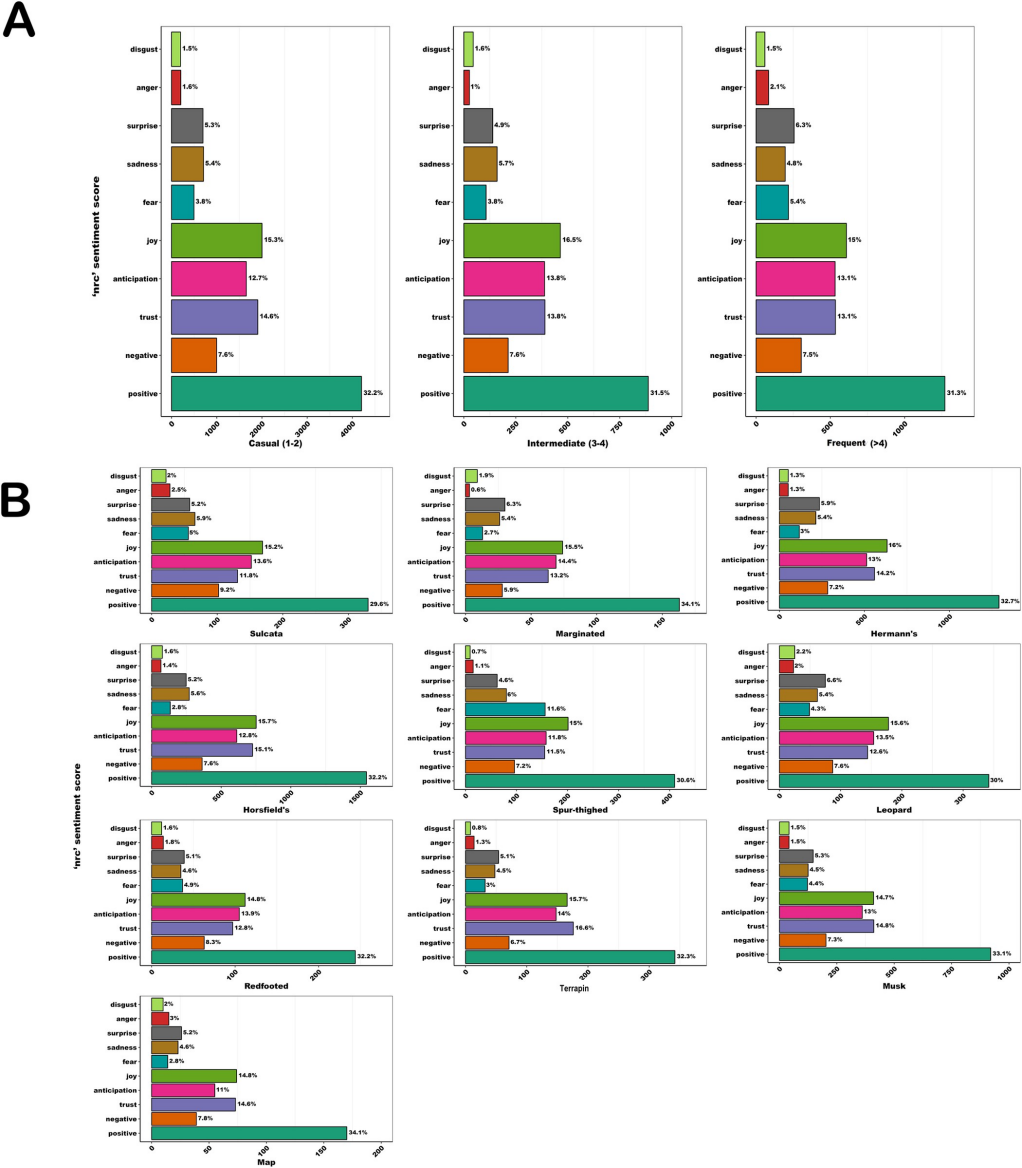
Mean *nrc* sentiment scores in advertisements categorised by A) seller-category, and B) species-type.

*Afinn* sentiment analysis scores suggest that frequent sellers use more positive language in their adverts than both casual and intermediate sellers (Fig 7A and 7B). *Afinn* scores across the 10 most advertised species-types indicate that the language used in adverts related to map turtles, musk turtles, Horsfield and terrapins were the least positive. It is notable that the three categories of turtles all fall within the four species-types with the least positive language. *Nrc* analyses suggest that at the finer scale, the ranking of the frequency of language used was consistent across seller-types (Fig 8A and 8B). Similarly, among species-types there were very few differences regarding the relative use rankings of *nrc* lexicon divisions; in all bar one of the species-types, the four most commonly used categories were: positive, joy, anticipation, and trust. The exception was spur-thighed tortoises, for which words associated with ‘fear’ replaced ‘trust’ in the top four, possibly on account of their fearsome spurs.

## Discussion

Results presented here suggest that chelonian adverts, sourced from a widely used UK pet classified advertisement site, are largely posted by infrequent sellers advertising a small number of commonly available species. Of these, aquatic chelonians i.e., map turtles, musk turtles and terrapins (hereafter collectively called ‘turtles’), were significantly more likely to be sold in multiples; to be advertised at a lower price; and to be associated with less positive language-use within advert descriptions–in comparison with their terrestrial counterparts i.e., tortoises. There were no strong temporal effects regarding number of adverts posted, outside of a lull over the Christmas and New Year period, suggesting that website traffic was driven by factors other than species-specific seasonal breeding. Combined, these results suggest that classified adverts, via this avenue, are not a significant driver of sales. Instead, our findings may reflect broader trends within the UK chelonian trade. For example, these results are consistent with this site largely being used as a route for chelonian owners to resell their animal(s), if they cannot, or do not wish to, keep them any longer. There are multiple lines of evidence supporting this hypothesis. First, the three most advertised species reported here, match the UK popularity rankings with regards to the most kept chelonians (in descending order: Hermann’s tortoises and Horsfield tortoises and musk turtles [41]). Second, the relative prices of the cheapest species-types on the platform (i.e., turtles) and the most advertised tortoises (Hermann’s and Horsfield) fall well below the recommended retail price per animal of those species as advertised in trade information (*Pers Comm Rendle*), suggesting they are not being sold for profit. In particular, the prices of turtles showed relatively little variation compared to the majority of tortoise species-types advertised on the site. Finally, most adverts were placed by casual sellers, i.e., those posting a maximum of two adverts per year. Combined, the high volume of infrequent sellers, the common species-types being sold, and the relatively low price point at which they were advertised, are consistent with the resale of animal(s), rather than larger-scale, profit-driven sales.

Specifically, these data suggest that turtles (specifically musk turtles, map turtles, and terrapins) are pets that have been bought for low prices, in large numbers, and are no longer wanted. Within some taxa these could be called ‘tank-busters’ [42] and long-lived, large species have been strongly associated with the likelihood of release into wild environments [43]. This tendency is likely enhanced by a couple of factors that are unique to turtles compared to tortoises. First, is the ease of purchase and low price of turtles in the UK, with their sale being common in garden centres and aquatics shops (i.e., those largely selling fish and other aquatic organisms). Second, are their husbandry needs, which are more onerous than commonly kept tortoises due to their requirements for a clean aquatic environment in addition to UV-B and correct heating. Another indicator of the different dynamics of sales within the chelonian taxon is highlighted by the different language used in the adverts between tortoises and turtles. Language used within turtle adverts was found to be less positive than those published for tortoises, perhaps highlighting different drivers of sale and attitudes of sellers/current owners. This suggests that, even within a relatively small clade, variation in motivations for sales, and human attitudes towards species-types, exists. Consequently, if we wish to develop human behaviour change interventions within the exotic trade, we must ground these processes within specific time points in the supply chain for a given species-type. Only by keeping species and breeds at the forefront of planned interventions, will we effectively target conservation or welfare concerns within the exotic trade.

The major narrative surrounding the exotic pet trade within scientific media is the risk it can pose to the conservation status of the species within that trade [44,45, but see 46]. The trade in both CITES-listed and non-CITES listed species is considerable, and data suggests that both can be a cause of serious concern for the conservation of biodiversity. This may be directly, by removing animals from the wild [23], or indirectly. Indirectly, biodiversity can be negatively affected via invasive species [47], spread of infectious pathogens to both native species [48] and potentially humans [49], or via increased trade of illegally sold animals under the auspices of legal trade (e.g., wild-caught rather than captive-bred animals; [50]. Data presented here suggest that online sales via this site pose a relatively small direct conservation risk, as the majority of adverts contained commonly-kept, widely traded chelonian species. However, one of the most commonly traded species: Hermann’s tortoise (*Testudo hermannii*), is listed on CITES Appendix II Annex A, and require permits for their sale in the UK [51]. In total, only 16% (92/569) of adverts for Hermann’s tortoise contained one of our search terms aimed at identifying the presence of correct permit and licensing for sale of the species. Whilst this may be due to a lack of knowledge, an oversight within the text of the advert description, or a methodological issue regarding search terms (e.g., they were not fully comprehensive) it could also be explained by a lack of correct permits for the tortoises being sold online. Quantifying the legal compliance of advertisements for species that are subject to trade restrictions (e.g., *Testudo hermannii*, but also *Trachemys* species due to their invasive potential) would add to our understanding of how to ensure transparency and good practice within the trade. One possibility would be to incorporate ethnozoological approaches to obtain data on legal compliance within online adverts, and more general information on the legality of practices within the hobby via private messages and groups (as in [8]).

Conservation status aside, the welfare of animals traded, or kept for any purpose is often overlooked as a concern, including in peer-reviewed literature [52]. Lack of coverage notwithstanding, the welfare of a sentient individual is important, regardless of the purpose for which it is being traded, be it food, products, or companionship. Some larger e-commerce and classified advertisement sites in the UK have voluntarily agreed to adhering to a ‘Code of Practice’ or set ‘Minimum Standards’ (e.g., PAAG https://paag.org.uk). These are, in theory, straightforward to implement and monitor. However, how this theory translates to practice remains unclear, and concerns persist across all animals advertised online, even in companion animals subject to greater regulations than reptiles [53].

The difficulty of monitoring online platforms has resulted in calls for tighter legislation [54] or even bans of online sales. If this were to happen within the UK chelonian trade, as highlighted by our findings, the essential question is: what would happen to these animals if online sales platforms no longer existed? This pertinent question remains unanswered. If casual sellers cannot advertise their animals, or are required to be licensed to do so (as suggested in recent a veterinary profession policy statement [54]), what will they choose to do? Given the increasing presence of alien individuals and invasive populations of Chelonia around the world [24], as well as reportedly large numbers of individuals being relinquished or abandoned (Ferguson *pers comm*), it seems likely that more owners may follow this course of action, or worse, should the option to resell their animal be removed without contingencies. Perhaps this suggests that if the number of animals being traded and their general welfare is a concern in this taxon, interventions would be better placed at points earlier in the supply-chain than online classified adverts, which may be perceived to be a useful place to start simply based on their high visibility and ease-of-access.

Our results and conclusions regarding the online trade contrast with some of those obtained from other geographic regions and taxonomic groups, highlighting the need for a cautious approach to transferring information from one location or taxon to another. For example, the volume of individuals, the diversity of taxa and the legality of the online trade can vary greatly. These factors will respond to supply and demand but will also depend on the geographic location of the country, its own diversity of species, and the cultural attitudes of that country towards utilisation of animals and their products. For example, Japan’s location between Asia and the Americas has been used to explain its importance in the reptile trade and the diversity of species that are available via online platforms [20]. Similarly, megadiverse countries may have a richer, more nuanced relationships with the natural world and its resources than the UK, which may result in concerns about the conservation of risk posed by the online trade [8,55]. Clearly, acknowledging cultural, economic, and social roles of animals in the society being studied, and engagement with a broad range of stakeholders are key in efforts to increase the ethical standing and sustainability of the pet trade.

Whilst the absolute number of chelonians for sale in our study available are not high relative to other regions (e.g. China [25] and USA [45]), trade prices suggest that they represent a relatively small proportion of the total number sold in the first point of sale (e.g. shops, aquatics centres). While the proportion of species-types in the trade may be accurately reflected by the data presented here, the scale of the trade and hobby of chelonian keeping would be under-estimated. This matters if we want to gain a better understanding of the overall UK market-chain in this group of species. Understanding the supply chain of exotic pets requires novel approaches and quantitative estimates of traffic at multiple points in the chain [56]. Our study provides a detailed example of how we could start to generate these estimates for a manageable platform for a focal taxon, in a specific region, at a given point in time. It seems unlikely that findings on the trade in Chelonia will be transferable to other taxa. However, similar methodological approaches incorporating other platforms/websites and taxa could quickly provide quantitative estimates of the number of animals being advertised and sold via e-commerce and classified advertisement sites. For example, including a broader range of specialist sales sites in addition to generalist classified advert sites could provide more insight into the diversity of species sold, their provenance, and the nature of sellers and buyers. In doing so, we would obtain a more detailed picture of the dynamics within the supply chain. Similar approaches at other points in the supply chain, incorporating both online and in person sales, may help to identify the scale and structure of chelonian trade in the UK as well as highlight some negative and positive impacts it may have.

Where time is a limitation, it would be useful to use rarefied sampling approaches [57] to identify the least amount of data needed to obtain robust estimates of the diversity and magnitude of the trade for a specific region and trade platform. Temporally, one of the concerns with this study may be that the data were collected over the year following the UK Covid-19 pandemic lockdown, and several studies have highlighted an increase in trade in more traditional companion animals during that time [58]. However, it is perhaps unlikely that we would see these effects within the timeframe this study. Our study began in July 2020: three months after the first lockdown in the UK (23^rd^ March 2020) and continued for 12 months. While the rate of demand and purchase for some companion animals (e.g., ‘pandemic puppies’ [59] and general convenience goods (e.g., toilet rolls [60]) increased rapidly during lockdown, it seems unlikely that chelonian pets followed this trend. Certainly, the absence of change in the number of adverts per day over the course of the study suggests that the effects of the pandemic on this taxon were not felt on this timescale.

The future of the exotic pet hobby in the UK is under much scrutiny. There has been a call for change, at several levels, from a number of stakeholders, including governmental committees [61], professional bodies [54], scientific researchers [62], registered charities [63], and popular media. Many potential interventions have been suggested, including increased and more efficient monitoring of non-CITES species [45], positive lists of only those species permitted to be kept as pets [64], and broader restrictions on the animals being kept and the people who are able to keep them [54,63]. However, the evidence-base for the efficacy of such measures is lacking. Ultimately, issues related to the trade and hobby are human-based and they require fostering of sustainable behaviours. Inadequately incorporating human behaviours, attitudes, and cultural values [55,65] and the capability, opportunity, and motivation of stakeholders to comply with interventions will likely severely restricts the ability of those interventions to meet their objectives [66]. If stakeholders in the UK trade of exotic pets do wish to improve animal welfare within the trade and hobby, it is therefore important that interventions are aimed towards the right issue, at the right time, at the right point in the supply chain. While dedicated classified advertisement sites provide a highly visible part of that trade, in the case of chelonians the species advertised and the reasons behind their sale represent the broader pet trade. As such, those websites may be unlikely to be an effective site for intervention within this clade of reptiles.

While highly visible and easily accessible, online classified advert platforms in the UK may reflect the broader trade within the country. Our results suggest that this website was mainly used to sell animals that people no longer wanted, rather than new sales for profit. As such, if any interventions were deemed to be necessary, they should perhaps be aimed earlier in the supply chain. Further, interventions should be species or taxon specific: turtles were sold more frequently by casual users than tortoises at significantly lower prices, using less positive language. This finding is consistent with a perception that turtles are a more disposable pet than tortoises, suggesting that perhaps they are a suitable target for further recommendations and interventions related to their sales and husbandry.

## Supporting information

S1 Table Total adverts per species-type posted between July 2020 and June 2021 (i.e., study period). (DOCX)

S2 Table Analyses of deviance table for the best fitting model of adverts placed per month. (DOCX)

S3 Table *Post hoc* Dunn’s tests on differences in frequency of advert placement among months of the year. (DOCX)

S4 Table *Post hoc* Dunn’s tests on differences in frequency of advert placement among the top ten advertised species-types. (DOCX)

S5 Table *Post hoc* Dunn’s tests on differences in frequency of advert placement among different categories of seller types. (DOCX)

S6 Table Frequency of adverts placed per seller over the year on the site. (DOCX)

S7 Table *Post hoc* Dunn’s tests on differences in frequency of placement of adverts including multiple individuals per advert in the top ten advertised species-types. (DOCX)
