# Supplementary material for: Online classified adverts reflect the broader United Kingdom trade in turtles and tortoises rather than drive it
Source: PLoS One. 2023 Jul 13;18(7):e0288725. doi: 10.1371/journal.pone.0288725 (PMC10343072; doi:10.1371/journal.pone.0288725)
Supplement: S1 Table — (DOCX) [file pone.0288725.s001.docx]

**S1 Table: Total adverts per species-type posted between July 2020 and June 2021 (i.e., study period).** Some will represent more than one individual for sale.

| Species | Total adverts |
| --- | --- |
| Horsfield tortoise | 666 (23.3%) |
| Hermann’s tortoise | 569 (19.92%) |
| Musk turtle | 435 (15.23%) |
| Terrapin | 178 (6.23%) |
| Spur-thighed tortoise | 175 (6.13%) |
| Sulcata | 152 (5.32%) |
| Leopard tortoise | 150 (5.25%) |
| Red-footed tortoise | 106 (3.71%) |
| Map turtle | 83 (2.91%) |
| Marginated tortoise | 70 (2.45%) |
| *Reeves’ turtle* | *44 (1.54%)* |
| *Cooter* | *42 (1.47%)* |
| *Indian star tortoise* | *31 (1.09%)* |
| *Snapping turtle* | *25 (0.88%)* |
| *European pond turtle* | *20 (0.7%)* |
| *Mud turtle* | *15 (0.53%)* |
| *Softshell turtle* | *13 (0.46%)* |
| *Sidenecked turtle* | *12 (0.42%)* |
| *Striped Chinese turtle* | *12 (0.42%)* |
| *Other unidentified/non-specified* | *8 (0.28%)* |
| *Pancake tortoise* | *8 (0.28%)* |
| *Painted turtle* | *7 (0.25%)* |
| *Radiated tortoise* | *7 (0.25%)* |
| *Fly river turtle* | *5 (0.18%)* |
| *Wood turtle* | *4 (0.14%)* |
| *Aldabran tortoise* | *3 (0.11%)* |
| *Box turtle* | *3 (0.11%)* |
| *Diamondback terrapin* | *2 (0.07%)* |
| *Egyptian tortoise* | *2 (0.07%)* |
| *Japanese pond turtle* | *2 (0.07%)* |
| *African helmeted turtle* | *2 (0.07%)* |
| *Fly-river turtle* | *1 (0.04%)* |
| *Mata mata* | *1 (0.04%)* |
| *Pink bellied turtle* | *1 (0.04%)* |
| *Snake necked turtle* | *1 (0.04%)* |
| *Spotted turtle* | *1 (0.04%)* |
