# Supplementary material for: Online classified adverts reflect the broader United Kingdom trade in turtles and tortoises rather than drive it
Source: PLoS One. 2023 Jul 13;18(7):e0288725. doi: 10.1371/journal.pone.0288725 (PMC10343072; doi:10.1371/journal.pone.0288725)
Supplement: S2 Table — (DOCX) [file pone.0288725.s002.docx]

**S2 Table: Analyses of deviance table for the best fitting model of adverts placed per month.**

LR Chisq Df p-value

Species-type 1310.98 9 <0.001

Month advert placed 45.12 11 <0.001

Seller-category 1125.44 2 <0.001

Species-type*Seller-category 114.05 18 <0.001

Null deviance: 2754.40 on 326 degrees of freedom

Residual deviance: 269.96 on 286 degrees of freedom

AIC: 1472.5
