# Supplementary material for: Online classified adverts reflect the broader United Kingdom trade in turtles and tortoises rather than drive it
Source: PLoS One. 2023 Jul 13;18(7):e0288725. doi: 10.1371/journal.pone.0288725 (PMC10343072; doi:10.1371/journal.pone.0288725)
Supplement: S4 Table — (DOCX) [file pone.0288725.s004.docx]

**S4 Table: *Post hoc* Dunn’s tests on differences in frequency of advert placement among the top ten advertised species-types.** Values correspond to p-value of comparison between months. Bold entries correspond to p<0.05 with the species-type in the column being more frequently advertised than the species-type in the row.
