# Supplementary material for: Online classified adverts reflect the broader United Kingdom trade in turtles and tortoises rather than drive it
Source: PLoS One. 2023 Jul 13;18(7):e0288725. doi: 10.1371/journal.pone.0288725 (PMC10343072; doi:10.1371/journal.pone.0288725)
Supplement: S5 Table — (DOCX) [file pone.0288725.s005.docx]

**S5 Table: *Post hoc* Dunn’s tests on differences in frequency of advert placement among different categories of seller types.** Values correspond to p-value of comparison between months. Bold entries correspond to p<0.05.
