# Supplementary material for: Online classified adverts reflect the broader United Kingdom trade in turtles and tortoises rather than drive it
Source: PLoS One. 2023 Jul 13;18(7):e0288725. doi: 10.1371/journal.pone.0288725 (PMC10343072; doi:10.1371/journal.pone.0288725)
Supplement: S6 Table — (DOCX) [file pone.0288725.s006.docx]

**S6 Table: Frequency of adverts placed per seller over the year on the site.**

| **Number of adverts placed** | **Frequency** |
| --- | --- |
| 1 | 1211 |
| 2 | 528 |
| 3 | 262 |
| 4 | 122 |
| 5 | 109 |
| 6 | 71 |
| 7 | 48 |
| 8 | 60 |
| 9 | 12 |
| 10 | 28 |
| 11 | 13 |
| 12 | 11 |
| 13 | 26 |
| 16 | 15 |
| 22 | 1 |
| 23 | 4 |
| 28 | 10 |
| 31 | 31 |
| 52 | 22 |
