# Supplementary material for: Online classified adverts reflect the broader United Kingdom trade in turtles and tortoises rather than drive it
Source: PLoS One. 2023 Jul 13;18(7):e0288725. doi: 10.1371/journal.pone.0288725 (PMC10343072; doi:10.1371/journal.pone.0288725)
Supplement: S7 Table — (DOCX) [file pone.0288725.s007.docx]

**S7 Table: *Post hoc* Dunn’s tests on differences in frequency of placement of adverts including multiple individuals per advert in the top ten advertised species-types.** Values correspond to p-value of comparison the pairwise comparison. Bold entries correspond to p<0.05 with the arrow direction (↑ or ↓) representing the frequency of multiple-individual adverts in the row species-type relative to the column species-type.
